# A comprehensive analysis of genetic risk for metabolic syndrome in the Egyptian population via allele frequency investigation and Missense3D predictions

**DOI:** 10.1038/s41598-023-46844-z

**Published:** 2023-11-22

**Authors:** Mahmoud Bassyouni, Mohamed Mysara, Inken Wohlers, Hauke Busch, Maha Saber-Ayad, Mohamed El-Hadidi

**Affiliations:** 1 Bioinformatics Group, Center for Informatics Sciences (CIS), School of Information Technology and Computer Science (ITCS), Nile University, Giza, Egypt; 2 Bioscience Research Laboratories Department, MARC for Medical Services and Scientific Research, 6th of October, Jiza, Egypt; 3 Microbiology unit, Belgian Nuclear Research Centre (SCK CEN), Mol, Belgium; 4 Medical Systems Biology Division, Lübeck Institute of Experimental Dermatology, and Institute for Cardiogenetics, University of Lübeck, Ratzeburger Allee 160, 23562 Lübeck, Germany; 5 Biomolecular Data Science in Pneumology, Research Center Borstel, 23845 Borstel, Germany; 6 University Cancer Center Schleswig-Holstein, University Hospital of Schleswig-Holstein, Campus Lübeck, 23538 Lübeck, Germany; 7 Department of Clinical Sciences, College of Medicine, University of Sharjah, 27272, Sharjah, UAE; 8 Pharmacology Department, College of Medicine, Cairo University, Cairo, 12613 Egypt; 9 Institute of Cancer and Genomic Sciences, College of Medical and Dental Sciences, University of Birmingham Dubai Campus, Dubai, United Arab Emirates

**Keywords:** Population genetics, Genetic variation

## Abstract

Diabetes mellitus (DM) represents a major health problem in Egypt and worldwide, with increasing numbers of patients with prediabetes every year. Numerous factors, such as obesity, hyperlipidemia, and hypertension, which have recently become serious concerns, affect the complex pathophysiology of diabetes. These metabolic syndrome diseases are highly linked to genetic variability that drives certain populations, such as Egypt, to be more susceptible to developing DM. Here we conduct a comprehensive analysis to pinpoint the similarities and uniqueness among the Egyptian genome reference and the 1000-genome subpopulations (Europeans, Ad-Mixed Americans, South Asians, East Asians, and Africans), aiming at defining the potential genetic risk of metabolic syndromes. Selected approaches incorporated the analysis of the allele frequency of the different populations’ variations, supported by genotypes’ principal component analysis. Results show that the Egyptian’s reference metabolic genes were clustered together with the Europeans’, Ad-Mixed Americans’, and South-Asians’. Additionally, 8563 variants were uniquely identified in the Egyptian cohort, from those, two were predicted to cause structural damage, namely, CDKAL1: 6_21065070 (A > T) and PPARG: 3_12351660 (C > T) utilizing the Missense3D database. The former is a protein coding gene associated with Type 2 DM while the latter is a key regulator of adipocyte differentiation and glucose homeostasis. Both variants were detected heterozygous in two different Egyptian individuals from overall 110 sample. This analysis sheds light on the unique genetic traits of the Egyptian population that play a role in the DM high prevalence in Egypt. The proposed analysis pipeline -available through GitHub- could be used to conduct similar analysis for other diseases across populations.

## Introduction

Egypt is one of the top ten countries with the highest prevalence of diabetes mellitus, according to the International Diabetes Federation (IDF). The number of diabetic patients in the Middle East and North Africa (MENA) is projected to increase from 34.6 million in 2013 to 67.9 million in 2035, with a 96% increase. In Egypt, individuals between the age of 20 and 79 years have a diabetes prevalence of around 20.9%, and the disease accounted for 122,684 deaths in the year 2021as reported in the IDF Atlas 10th edition 2021^1^. In the same report, the IDF calculated that 10.93 million Egyptians reported having diabetes, while 6.8 million have undiagnosed form of the disease. In addition, estimates suggest that 62% of Egyptians with diabetes and the majority of those with prediabetes are probably undiagnosed. The dramatic rise in diabetes prevalence in Egypt from around 7.3 million in 2011 to 10.9 million in 2021 over a very short period is concerning, especially that by the year 2045, it is anticipated that this number would increase to 19.9 million. Apart from being a significant public health issue, diabetes is predicted to have cost the Middle East area $13.6 billion in 2013 (14% of its overall health care expenditures), which represents just 2.5% of the disease's global expenditures. The economic burden of Type 2 Diabetes (T2D) in Egypt was assessed by cost experts to be $1.29 billion in 2010, not considering expenses connected to prediabetes or lost productivity that is likely to be doubled by the year 2030^2^.

Several risk factors have been attributed to the high prevalence of diabetes—particularly in Egypt- including physical inactivity and obesity, particularly visceral adiposity^2^. The Egypt National STEPwise Survey for Non-communicable Diseases Risk Factors Report (2017), a national household survey on citizens aged 15–69 years old, reported that approximately 35.7% of adults were obese (BMI > 30 kg/m^2^), with a prevalence of 48.8% among women and 24.8% among men^3^. In the same report, it has been shown that obesity percentage increased from 31.3% at 2012 and reached 35.7% in 2017. However, according to a more recent survey -100 million health survey- conducted in Egypt in 2019 including 49.7 million adult Egyptian citizens (≥ 18 years old), 39.8% suffered from obesity (BMI ≥ 30 kg/m^2^). Prevalence of obesity in females was revealed to be more than males from the same age group, with 49.5% for adult females compared to 29.5% of males^4^.

A group of disorders known as metabolic syndrome increases the chance of developing heart disease, stroke, and type 2 diabetes. Hypertension, Obesity, and Hyperlipidemia are among these problems. According to Abd Elaziz et al., a high metabolic syndrome prevalence of 55% was found in Egyptians, 85.6% among diabetics, and 76.6% among hypertensive patients^5^. The prevalence of hypertension in Egypt is notably high, while the rates of awareness, treatment, and control are comparatively low. The coexistence of other cardiovascular risk factors exacerbates hypertension in 60% of patients, leading to heightened cardiovascular morbidity and mortality^6^. According to the World Health Organisation (WHO) 2020 hypertension Egypt report, it is estimated that 17.8 million of the Egyptian population in 2017 had hypertension, with 15.4 million not having it under control^7^. Furthermore, the occurrence of dyslipidemia exhibited variability amongst the overall population of Egypt, with a range of 19.2–36.8%. However, the incidence of dyslipidemia was comparatively greater in individuals diagnosed with acute coronary syndrome (ACS) at 50.9 and 52.5%, and those with coronary artery disease at 58.7%. According to a national report, dyslipidemia screening was conducted on 8.6% of the overall population. However, there is a lack of data regarding the rates of diagnosis and treatment^8^.

The high prevalence of DM and the other metabolic traits are characterized by a wide range of DNA sequence variants, that play a role in phenotypical/pathophysiology of the diseases^9^. High-impact variants, with allele frequencies below 0.5%, cause monogenic and syndromic metabolic diseases like MODY and familial hypercholesterolaemia^10,11^. These diseases typically appear early in life and have a clear familial pattern. Late-onset metabolic diseases, on the other hand, are primarily influenced by common variants, with allele frequencies greater than 5%. Genome-wide association studies have identified hundreds to thousands of these common variants, each with a minor effect on disease risk^12–15^. Recent research with larger sample sizes and sequence data has shown that the genetic predisposition to prevalent metabolic traits in later life is primarily due to a broad distribution of common allele effects that gradually decrease in impact^16,17^.

In this work, we aim to assess the potential genetic risk of metabolic syndrome and identify the commonalities and distinctive genetic traits between the Egyptian and the 1000-genome subpopulations (Europeans, Ad-Mixed Americans, South Asians, East Asians, and Africans). For this purpose, the metabolic syndrome using the allele frequency of different populations’ variations, supported by genotypes’ principal component analysis, was assessed. Additionally, the analysis steps are presented as a single pipeline -through GitHub- to analyse similar diseases among various populations.

## Results

### Intersection analysis of diabetes type II with obesity, hypertension and hyperlipidemia: annotation and characterization of associated genes

**Figure 1:**
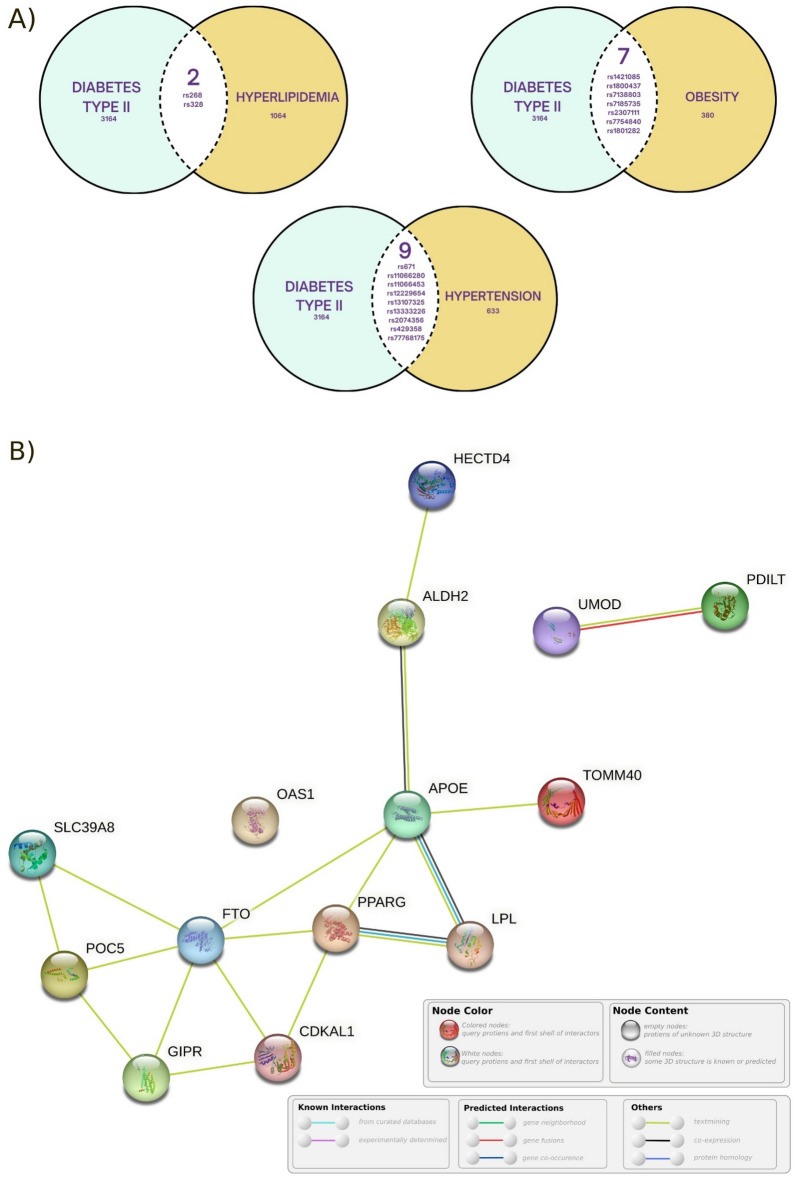
Protein-Protein Interaction network analysis from STRING database and the intersection results of the metabolic diseases variants with T2D. (**A**) Venn Diagrams represent the genetic overlap between T2D and each of Hyperlipidemia, Obesity, and Hypertension as -collectively- the figure illustrates the shared variants between these conditions. Two variations are shared between T2D and Hyperlipidemia. In a similar fashion, Obesity and Hypertension share 7 and 9 variants, respectively. (**B**) Genes network analysis created by STRING database, showing the possible interactions between each and every protein coding gene. The light green colour edge that nearly links every two adjacent nodes refers to the text mining legend which means that they are co-mentioned in PubMed Abstracts. More edges consequently mean stronger relationships between the two genes.

Here we explore the genetic overlap between T2D, Hyperlipidemia, Obesity, and Hypertension. Using the search criteria described in the methods, we were able to identify 4 datasets from Ensembl. In total 3164, 633, 380, and 1064 single nucleotide variants were found associated with T2D, Hypertension, Obesity, and Hyperlipidemia, respectively. A total of 18 Single Nucleotide Variants (SNVs) were shared between the T2D and the other disorders. Among these, two SNVs were shared with Hyperlipidemia, seven SNVs were shared with Obesity, and nine SNVs were shared with Hypertension (Fig. 1A). Those 18 variants were identified to be linked to various genes using the Variant Effect Predictor (VEP) tool from Ensembl. When analyzing the impact of these variants on upstream and downstream effects, we found that they affect a total of 19 genes, 14 of which were protein coding (Supplementary Table S1), while the other five were pseudogenes and RNA genes (Supplementary Table S2). Results of the Hypergeometric tests of the STRING database network analysis for the protein coding genes (Fig. 1B) showed significant interactions with Protein–Protein Interaction (PPI) enrichment p-value of 3.92 × 10^–12^ with most of the disease-gene associations be T2D, Familial Hyperlipidemia, and Acquired Metabolic Disease.

Since all the shared variations were single nucleotide variants (SNVs), the first intersection (Fig. 1A), yielded the variants ***rs268*** and ***rs328***. These variants are associated with the LPL gene and exhibit missense and stop-gained effects, respectively (Table 1). The second intersection included the variants ***rs1421085***, ***rs1800437***, ***rs7138803***, ***rs7185735***, ***rs2307111***, ***rs7754840***, and ***rs1801282***. Among these variants, ***rs1421085*** and ***rs1800437*** correspond to the FTO and GIPR genes, respectively, and are characterized as intronic and missense variants. The remaining variants in this intersection are found in intergenic regions or exhibit intronic or missense effects. The third intersection, which involved Hypertension, encompassed the variants ***rs671***, ***rs11066280***, ***rs11066453***, ***rs12229654***, ***rs13107325***, ***rs13333226***, ***rs2074356***, ***rs429358***, and ***rs77768175***. Notably, ***rs671, rs13107325***, and ***rs429358*** are associated with the ALDH2, SLC39A8, and APOE genes -respectively- and represent missense variants. The other variants in this intersection are either intronic or of intergenic nature. These identified variants provide further insights into the genetic junctions between T2D and the aforementioned metabolic diseases.

**Table 1:** Provides the list of intersected variants' annotation along with relevant details about each one.

Variant ID	Gene	Consensus	Position	Allele	Global MAF
rs11066280	HECTD4	Intron variant	chr12:112379979	T > A/T > G	0.04 (A)
rs11066453	OAS1	Intron variant	chr12:112927816	A > G	0.025 (G)
rs12229654	Intergenic variant		chr12:110,976,657	T > G	0.032 (G)
rs13107325	SLC39A8	Missense variant	chr4:102267552	C > A/C > T	0.024 (T)
rs13333226	UMOD	Intron variant	chr16:20354332	A > G	0.024 (G)
rs1421085	FTO	Intron variant	chr16:53767042	T > C	0.23 (C)
rs1801282	PPARG	Missense variant	chr3:12351626	C > G/C > T	0.07 (G)
rs2074356	HECTD4	Intron variant	chr12:112207597	G > A	0.026 (A)
rs2307111	POC5	Missense variant	chr5:75707853	T > A/T > C	0.38 (T)
rs268	LPL	Missense variant	chr8:19956018	A > G	0.005 (G)
rs328	LPL	Stop gained	chr8:19962213	C > A/C > G	0.09 (G)
rs429358	APOE	Missense variant	chr19:44908684	T > C	0.15 (C)
rs671	ALDH2	Missense Variant	chr12:111803962	G > A	0.035 (A)
rs7185735	FTO	Intron variant	chr16:53788739	A > G/A > T	0.34 (G)
rs7754840	CDKAL1	Intron variant	chr6:20661019	G > A/G > C/G > T	0.4 (C)
rs77768175	HECTD4	Intron variant	chr12:112298314	A > G	0.032 (G)
rs7138803	Intergenic variant		chr12:49,853,685	G > A/G > T	0.26 (A)
rs1800437	GIPR	Missense variant	chr19:45678134	G > C	0.16 (C)

The variants are designated by their variant ID, and the genes to which they correspond are provided. In the consensus column, the type of variation, such as Intron Variant, Missense Variant, or Stop Gained, is indicated. In the Position column, the chromosomal location of the variant is specified. The Allele column displays the observed allele variations, including the reference and alternative alleles. The Global MAF column displays the results from the 1000 Genomes Project Phase 3.

### Genotypes PCA and heatmap analysis of allele frequencies matrix reveal population clustering

**Figure 2:**
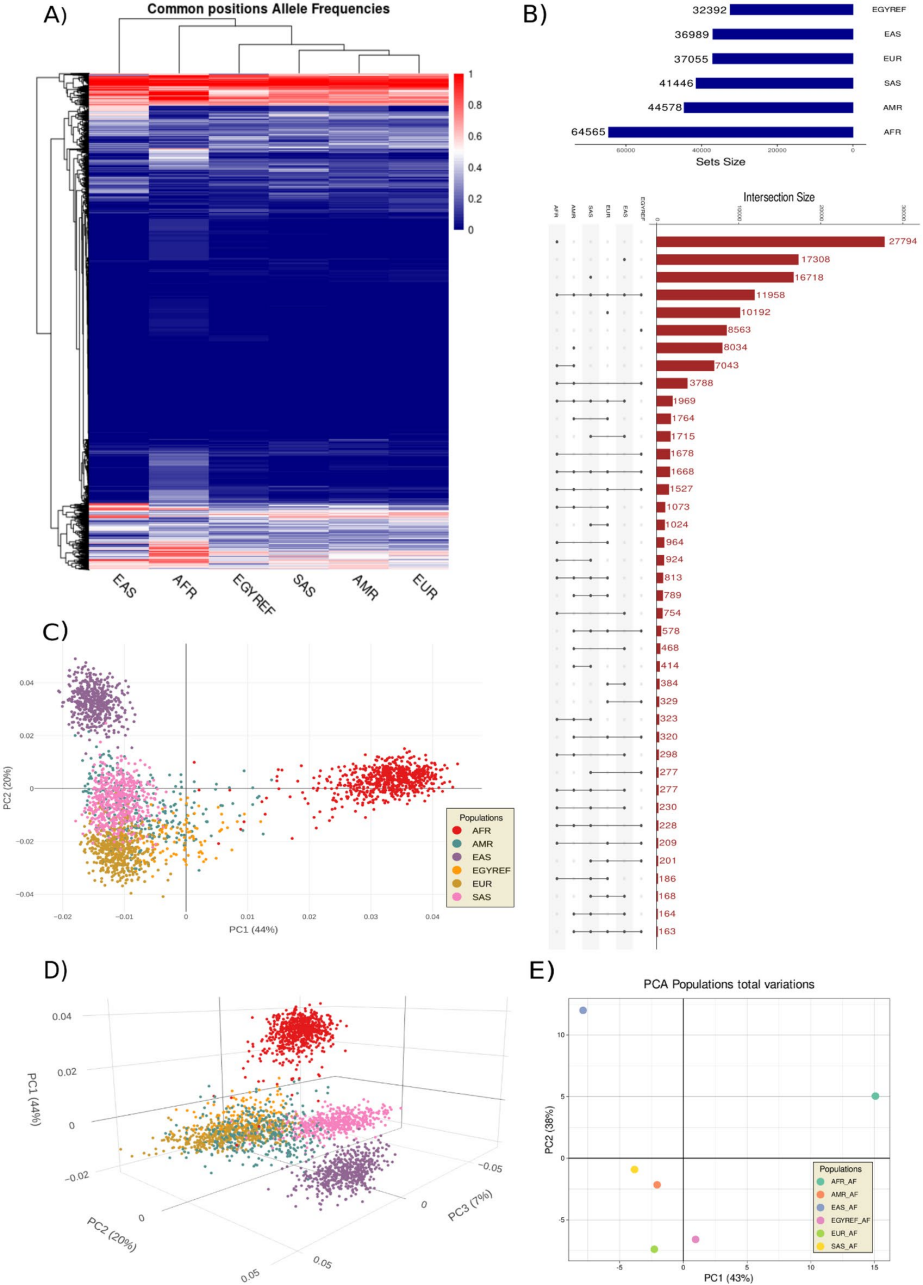
Genetic Variation of the metabolic syndrome genes Among Six Populations: Insights from Allele Frequencies and Genotypes. (**A**) and (**E**) Allele Frequencies (AF) Heatmap and Principal Component Analysis (PCA): In this panel, the analysis was based on the allele frequencies (AF) of shared variants. The PCA and heatmap analyses were conducted to investigate the relationships and clustering patterns among individuals. The AF heatmap (**A**) and principal component analysis (**E**) revealed distinct clustering patterns. Individuals of European, Ad-Mixed American, and South Asian descent clustered, while Egyptians demonstrated a subsequent tendency to cluster. Africans and East Asians formed distinct clusters, implying their genetic profiles are distinct. (**B**) The plot represents the genetic variation present in six distinct populations, namely Africans, Ad-Mixed Americans, East Asians, Egyptians, Europeans, and South Asians. The present study employed the UpSetR package to investigate the intersection of variants, thereby elucidating the count of distinct variants for every population. The dots within the matrix symbolize the active involvement of a specific population in an inclusive intersection. Meanwhile, the edges connecting these dots indicate the participation of other populations in this intersection. The height of the dots represents the number of elements within that set, with taller columns indicating larger sets. (**C**) 2D and (**D**) 3D Genotypes Principal Component Analysis (PCA) Visualization. Genotypes were pruned for MAF of 0.05 with indep-pairwise of 50 5 0.5. The consistent clustering patterns observed in the genotypes’ PCA and AF heatmap indicates a strong correlation. Particularly noteworthy is the grouping of Egyptian genotypes with those of Europeans, Ad-Mixed Americans, and South Asians, which suggests significant genetic similarities among these populations. In contrast, African genotypes exhibited separate clustering from both East Asians and the remaining genotypes, emphasizing the distinct genetic profiles of both Africans and East Asians. 2D and 3D HTML interactive plots can be found in the (Supplementary Files S1 and S2), respectively. A Bar plot for the proportional variances of the PCA is depicted in (Supplementary Fig. S2).

The examination of the metabolic genetic variants across the current range of populations encompassing Egyptians, East Asians, Europeans, South Asians, Ad-Mixed Americans, and Africans, elucidated interesting patterns and interrelationships. The upset (Fig. 2B) and Venn intersections (Supplementary Fig. S1) revealed that Africans had the largest number of unique variants with 27,794 followed by East and South Asians with 17,308 and 16,718, respectively. However, the European population showed 10,192 unique variants, Egyptians and Ad-Mixed Americans were the closest and the least in uniqueness with 8563 and 8034 with the latter being lesser. It is worth mentioning that all the sets shared 11,958 unique variants. Notably, the Egyptians demonstrated significant genetic overlap with Africans, with 1,678 variants in common. In contrast, a smaller number of common variants was observed between East Asians, Europeans, and South Asians, indicating a degree of genetic distinctiveness for Egyptians in relation to these populations.

The intricate genetic structure of the Egyptian population, suggesting both shared ancestry and potential unique evolutionary trajectories, is further emphasized by the analysis of intersection patterns. Notably, the intersection between Egyptians, Ad-mixed Americans, and Africans encompassed 3,788 variants, highlighting shared genetic components among these groups. Conversely, Egyptians exhibited limited intersections with East Asians, Europeans, and South Asians, indicating a relative genetic differentiation from these populations.

Unlike the variants intersection, the results obtained from the genotypes and allele frequencies principal component analysis (PCA) and heatmap demonstrated a noticeable clustering pattern, whereby individuals of European, Ad-Mixed American, and South Asian descent were observed to cluster together, with Egyptians exhibiting a subsequent clustering tendency. Distinct clusters were observed among individuals of African and East Asian ancestry. A heatmap and a PCA generated for the allelic frequency (Fig. 2A–E) data exhibited analogous clustering patterns to those observed in the genotypes PCA (Fig. 2C–D). The observed clustering of the Egyptians AF and genotypes with those of Europeans, Ad-Mixed Americans, and South Asians is noteworthy. Conversely, the genotypes of Africans and East Asians showed distinct genetic characteristic reflecting the complex interplay of genetic diversity, historical migrations, and population dynamics.

In summary, the analysis of variant set intersections, allele frequencies, and genotypes' principal component analysis reveals a contrasting picture in the genetic relationships involving Egyptians. While the variant set intersections indicate a substantial sharing of variants with Africans, the AF and PCA analyses demonstrate a closer clustering of Egyptians with Europeans, Ad-Mixed Americans, and South Asians. These contrasting results suggest a complex genetic landscape for Egyptians, characterized by both shared genetic affinity with Africans and genetic similarities with Europeans, Ad-Mixed Americans, and South Asians.

### Analysis of two protein-coding SNPs with structural damage potential in CDKAL1 and PPARG genes

To assess the potential functional and structural implications of protein coding variants that are unique to the Egyptian cohort, an analysis was conducted using the Missense3D database, in combination with protein structure data obtained from the AlphaFold database. The information regarding the VEP output and the filtered SNVs can be found in (Supplementary Table S3) and (Supplementary Table S4), respectively. Results of the analysis of a total of 60 protein-coding SNPs (Supplementary Table S5) predicted two SNVs in CDKAL1 and PPARG to be causing structural damage. It is noteworthy that both variants were identified as heterozygous in two Egyptian individuals out of a total of 110 Egyptians with a Minor Allele Frequency (MAF) of 0.0045 represented by a half genotype. However, to accurately determine the prevalence of the identified variants and rule out the potential for their rarity or exclusivity within the Egyptian population, it is imperative to obtain larger cohorts of individuals with Egyptian ancestry.

The detailed characteristics and outcomes of this analysis are presented in (Table 2). The table encompasses various information, including the chromosome and position of each SNV, the alternative allele observed, the gene symbol associated with the variant, existing variations as reported by the VEP, the protein position affected by the SNV, the resultant amino acid change, and the findings derived from the Missense3D analysis. Notably, the Missense3D analysis revealed specific consequences for each SNV, such as buried hydrogen bond breakage for the CDKAL1 variant (represented as rs756851756) at protein position 360 and altered cavity configuration for the PPARG variant at position 23, resulting in an amino acid change from alanine (A) to valine (V).

**Table 2:** Missensense3D structural damage prediction analysis result for the protein coding variants. Two SNPs found in two different samples out of the 110 Egyptian cohort with a minor allele frequency of 0.0045 were identified as potentially causing structural damage.

Chromosome	Position	Alt Allele	Gene symbol	VEP existing variation	Protein position	Amino acid change	Missense3D analysis result
6	2106570	T	CDKAL1	rs756851756	360	T/S	Buried H-bond breakage
3	12351660	T	PPARG		23	A/V	Cavity altered

These SNPs were found in the CDKAL1 and PPARG genes. The table includes the chromosome number, position, alternative allele, gene symbol, existing variation according to Variant Effect Predictor (VEP), protein position, amino acid change, and the results of Missense3D analysis.

**Figure 3:**
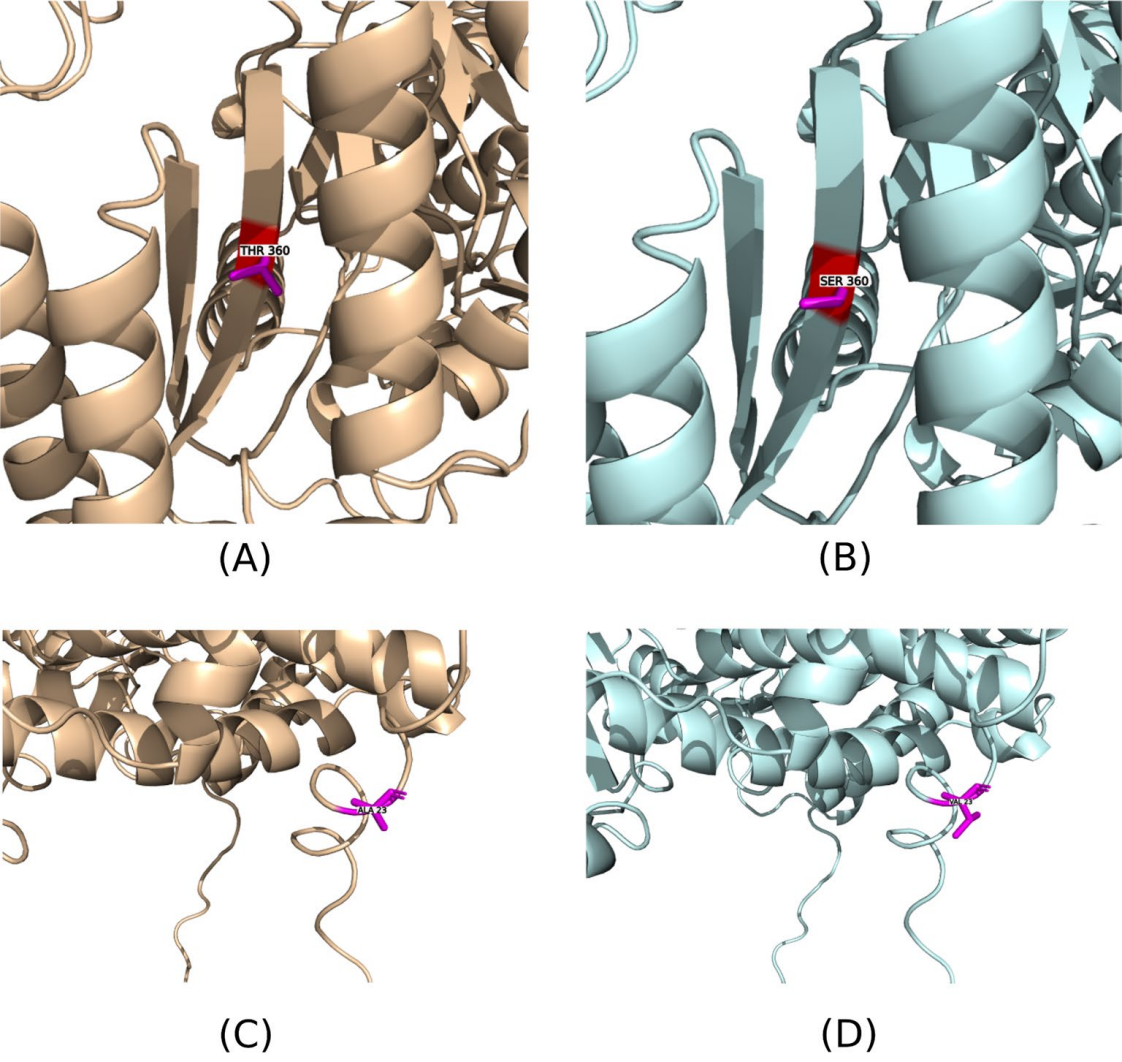
Missense3D Predicted damaged Structures for the mutant types compared to the wild ones. (**A**) Wild Type, (**B**) Mutant Type. Structural Damage predicted for the protein Threonylcarbamoyladenosine TRNA Methylthiotransferase (CDKAL1) as for the SNP rs756851756. This substitution disrupts all side-chain/side-chain H-bond. (**C**) and (**D**) Structural Damage predicted for the protein peroxisome_proliferator-activated_receptor_gamma (PPARG) as for the SNP 3_12351660_T. (**C**) Wild Type, (**D**) Mutant Type. The substitution leads to the contraction of cavity volume by 191.592 Å^3^.

The structural impact of these variants was further visualized through the comparison of wild-type and mutant structures of the respective proteins, as illustrated in (Fig. 3A–B) and Figure (Fig. 3C–D). The observed structural alterations, including the disruption of hydrogen bonds and contraction of protein cavities, provide crucial insights into the potential functional consequences of these protein-coding variants specific to the Egyptian population.

## Discussion and conclusion

Metabolic syndrome diseases including Hypertension, Hyperlipidemia, and Obesity are highly linked to T2D. Those are believed to be linked to genetic factors that are different across populations. In this work we are trying to identify the unique genetic traits in the Egyptian population that contribute to the high prevalence of metabolic syndrome and its connection with the T2D. Our analysis revealed that genes linking T2D with different other components of metabolic syndrome (namely: obesity, hypertension, and dyslipidemia) exhibit high number of SNPs in the Egyptian cohort. We will discuss the genes of interest at the pathophysiological level.

Our current study revealed that FTO variants are shared among diabetes and obesity. Certain genetic variants in the fat mass and obesity-associated (FTO), were reported to be associated with obesity^18,19^ in addition to their effect on food preference patterns such as increased total energy intake, in particular carbohydrate consumption^20,21^. The FTO gene was strongly linked with the development of obesity^22^. Among tens of loci identified to be associated with obesity, FTO gene has been identified as one of the influential genes with significant impact^23^. The FTO gene variants has been reported to associate with increased energy, fat, and protein intake^23,24^. The link of FTO and diabetes (and its complications) has been previously investigated. The study of Bego et al. revealed a significant association of FTO genetic variant rs8050136 A > C with the major markers of insulin resistance, obesity, and inflammation, in the population of West Balkan region^25^. Our previous study reported an association between FTO rs9939609 “A” allele and the impaired fasting glucose and insulin resistance in Emirati population^26^. Obesity and insulin resistance are clearly underlying the pathophysiology that leads to T2D.

Also, GIPR was shared between obesity and diabetes. The Gastric inhibitory polypeptide receptor (also called Glucose-dependent insulinotropic peptide) has been identified as one of two incretin hormones, linking nutrient intake to systemic metabolism^26,27^. The other incretin is Glucagon-like peptide-1 (GLP-1). Intriguingly, a paradox has emerged upon contradictory findings on the GIPR agonists versus antagonists as potential anti-obesity therapies. Targeting the pathways of endogenous nutrient-stimulated hormones allowed for better efficacy with acceptable safety, according to recent research using long-acting glucagon-like peptide-1 (GLP-1) receptor agonists^26,28^. Another attractive target is GIP, as it controls energy balance in the brain and adipose tissue via signalling through cell-surface receptors (GIP)^26,29^. An agonist that combines GIP and GLP receptor stimulation, Tirzepatide, has been recently approved by the FDA as a new treatment of diabetes mellitus type 2^30^. In addition, it led to sustained remarkable reductions in body weight, according to the SURMOUNT-1 trial, NCT04184622^31^. Our findings emphasize the link between obesity and diabetes through GIPR SNVs. We postulate that such SNVs may lead to GIP dysfunction, with impaired insulin secretion in response to the incretin effect in such patients.

Furthermore, peroxisome proliferator-activated receptor gamma is a nuclear receptor and drug target for insulin sensitizers and hypolipidemic agents. The encoded protein of *PPAR-γ* gene is a regulator of adipocyte differentiation. Moreover, *PPAR-γ* has been involved in the pathogenesis of obesity, diabetes, atherosclerosis, and cancer. A class of insulin sensitizers (thiazolidinediones) are basically agonists of *PPAR-γ*. PPAR-ligands support the storage of fatty acids in fat depots and control the expression of hormones released by adipocytes that affect glucose homeostasis. Improved insulin sensitivity is the result of the PPAR-ligands' pleiotropic activities^32^. In general, PPARs control the expression of genes involved in the inflammation through controlling different pathways of inflammatory response^33^. The link of obesity, T2D, inflammation and cancer has been thoroughly investigated and reported to involve, at least in part, PPAR and their associated pathways^34^.

In our analysis, LPL was the single gene shared between T2D and hyperlipidemia, with an obvious functional link. LPL gene encodes lipoprotein lipase; an enzyme expressed in the heart, muscle, and adipose tissue. LPL functions as a homodimer and has the dual functions of triglyceride hydrolase and ligand/bridging factor for receptor-mediated lipoprotein uptake. Interestingly, LPL mutations lead to a spectrum of LPL deficiency with the severest form known as type I hyperlipoproteinemia. An epidemiological study linked Lipoprotein lipase to insulin resistance, vitamin D and T2D in the Chinese^35^. Interestingly, Puri et al. found out that a significant metabolic reprogramming occurs when the diabetic heart cannot use lipoprotein lipase to control its own FA supply. This occurs with increasing diabetes severity and is linked to how the heart may handle excess fatty acids coming from adipose tissue. This change causes a cardiac metabolic profile that includes oxidative stress, triglyceride accumulation, mitochondrial FA excess, and cell death^36^.

Several genes were shared between T2D and hypertension. In a study by Andreassen et al., HECTD4 was identified as an independent Locus associated with systolic blood pressure and high LDL through conditional False Discovery Rate (FDR; < 0.01)^36^. HECTD4 SNPs were reported in association with alcohol consumption, with significant increased risk of type 2 diabetes^37^. Moreover, uromodulin is the most abundant protein in mammalian urine under physiological conditions. This protein may act as a constitutive inhibitor of calcium crystallization in renal fluids. Its excretion in urine may provide defence against urinary tract infections caused by uropathogenic bacteria. Uromodulin was reported to have a causal and adverse effect on kidney function, being involved in salt reabsorption via the NKCC2 (Na^ +^^−^ K ^+^^ −^ 2Cl ^−^ cotransporter) of the loop of Henle. Salt sensitivity is an important factor in the pathophysiology of hypertension^38^, and uromodulin may therefore represent a shared point of the common complications of diabetes and hypertension. In addition to this, mutations in the APOE gene result in type III hyperlipoproteinemia (familial dysbetalipoproteinemia), with impaired chylomicron and VLDL remnant clearance and consequent increased plasma cholesterol and triglycerides. APOE, hypertension and Diabetes significantly increase the risk of dementia, including Alzheimer’s disease^39,40^. A link has been established through the action of APOE4 that leads to endosomal entrapment of insulin receptors^41^. In addition to this, mutations in the APOE gene result in type III hyperlipoproteinemia (familial dysbetalipoproteinemia), with impaired chylomicron and VLDL remnant clearance and consequent increased plasma cholesterol and triglycerides. APOE, hypertension and Diabetes significantly increase the risk of dementia, including Alzheimer's disease^39,40^. A link has been established through the action of APOE4 that leads to endosomal entrapment of insulin receptors^41^.

Clustering for the AFs of the Egyptian Reference metabolic genes goes along with the same populations’ proximity in Wohlers et al.^42^. Intriguingly, South-Asians close stratification with the Europeans has been discussed before in C. Chambers et al.^43^ with the low genetic distance value “F_ST_”. Meanwhile, the PCA results confirm the same information with the Egyptian genotypes clustered nearly at the center with the European and closely relating to the Ad-Mixed American, and South-Asian ones. Interestingly, the African population’s genotypes clustered distinctly despite the high numbers of variants they shared with the Egyptian ones. The observed phenomenon may be attributed to the possibility that the common genetic variations shared by Egyptians and Africans could be localized in particular genomic regions that underwent minimal differentiation, thereby being eliminated during the linkage disequilibrium filtering process. Moreover, results from the allele frequency PCA showed distinctive characteristics of the African and East Asian population suggesting genetic divergence for both of them. These findings shed light on the multifaceted nature of human genetic diversity for the genes responsible for the metabolic syndrome, unveiling shared genetic traits and distinctive characteristics across diverse populations. Moreover, these findings emphasize the need for comprehensive investigations to unravel the underlying mechanisms driving these contrasting patterns and gain a deeper understanding of the genetic structure and history of the Egyptian population.

Finally, the effect of the predicted structural damage of the protein coding variants is noteworthy. Besides, the thermostability of proteins against denaturation is greatly influenced with the number of the H-bonds formed^44^. Wild type of the protein Threonylcarbamoyladenosine TRNA Methylthiotransferase shows Hydrogen bonding between the side chain 360 Threonine (THR) and the main chain 320 Isoleucine (ILE) with 2.75Å. Another bond happens to be between 360 THR and ASP 361 with 3.67 Å. While the at the main chain, 322 Valine (VAL) forms a hydrogen bond (H-bond) with the 360 THR with 2.89Å. Conversely, the prediction result didn’t find any H-bond at the mutant structure with the 360 serine (SER). Furthermore, it is worth noting that the SNP “rs756851756” showed uniqueness to the Egyptian Reference in our dataset, however in the ALFA Allele frequency it expresses an AF of 0.00007 with 1 European subpopulation allele out of 14,050^45^. Additionally, cavity contraction of the peroxisome proliferator-activated receptor gamma was predicted as for the SNP for the allele “T” at the position 12,351,660 on Chromosome 3, that happened to be causing amino acid change for the 23rd Alanine (ALA) to Valine (VAL). This change caused contraction for the cavity volume by 191.592 Å^3^. A biological validation is required to confirm the pathogenicity of such volume contraction.

In conclusion, our study identified several genes that contribute to the high prevalence of metabolic syndrome, particularly in the context of T2D, in the Egyptian population. In this study, we introduce a comprehensive pipeline to investigate the allele frequency variations among different populations and supported our findings through genotypic PCA analysis. Our results demonstrated close clustering of the reference metabolic genes of Egyptians with those of Europeans, Ad-Mixed Americans, and South Asians. Additionally, we identified 8563 variants unique to the Egyptian cohort. Further analysis of these distinctive variants unveiled two missense variants that were found to be heterozygous in two different samples out of the 110 Egyptian cohort, CDKAL1: 6_21065070 (A > T) and PPARG: 3_12351660 (C > T) and were predicted to cause structural damage according to Missense3D. These findings suggest that the reference metabolic genes of Egyptians may exhibit population-specific alterations, potentially impacting disease susceptibility and tailored therapeutic approaches. However, Larger cohorts of Egyptian ancestry are necessary to assess the prevalence of the variants and exclude the possibility that the detected variants are ultra-rare or private also in the Egyptian population. Moreover, the emerging field of precision medicine (PM) bears promise for combating Egypt's growing diabetes epidemic. Population-scale genetic, clinical, and lifestyle data can be analysed to develop targeted strategies for specific subgroups, thereby augmenting outcomes, extending lifespan, and decreasing healthcare expenditures. Through this analysis, we hope to pave the way for advancements in healthcare practises, enhanced disease management, and an overall improvement in Egypt's public health outcomes. Future work is warranted to explore the underlying factors contributing to the observed genetic similarities and differences among populations, providing valuable insights into the genetic history and relationships of the Egyptian population within the broader context of human metabolic genetic diversity.

## Methods

### Data collection and pre-processing

The datasets selection was based on searching the 1000-genome project Ensembl database, using the name of the diseases representing the components of the metabolic syndrome, namely, Type 2 Diabetes, Hypertension, Obesity, and hyperlipidemia. In the data availability section, the links to these four datasets were documented. The Ensembl database utilizes ontologies such as HPO (Human Phenotype Ontology) and EFO (Experimental Factor Ontology) to map and display disease phenotypes, facilitating data integration across domains^46,47^.

The common variants between Type II Diabetes and the other three diseases were identified and the Ensembl's Variant Effect Predictor (VEP) -release 109^48^ was employed to analyze the resulting list of common variants and identify the associated genes. The reported variants by VEP were filtered by the removal of duplicates and the dbSNP database -build 156^49^ was utilized to obtain the annotation of the common variants.

To annotate the protein-coding genes linked to the common variants and explore their potential interactions, the STRING database v11.5^50^ was employed. This allowed for the examination of possible networks among these genes. Additionally, the GeneCards database v5.15^51^ was used to annotate the genes while particularly focusing on identifying RNA genes and pseudogenes.

To collect information on the VEP shortlisted genes, we used the available variant data from an Egyptian Genome study^42^ which integrated previously generated raw data^52^. Several procedures were taken to prepare the genomic data Variant Calling Files (VCF) of the Egyptian and the 1000 Genome samples for analysis. These procedures included concatenating VCFs with BCFtools v1.16^53^, removing duplicate entries, normalizing multiallelic sites to biallelic sites, and extracting Allele Frequencies (AFs). Separate lists containing chromosome, position, and alternative allele information were generated for each subpopulation in the 1000 Genome sample. Similarly, pertinent data were extracted from the Egyptian genome VCF and stored in a separate list. Using these techniques, standardized formats of genetic variation within the genes of interest were generated, thereby facilitating insightful analysis. This approach allowed us to obtain insights into the common genetic variants associated with Type II Diabetes, Hyperlipidemia, Hypertension, and Obesity, thereby enhancing our understanding of the genetic basis of these complex diseases.

### Genetic analysis and visualization of populations

In this investigation, a genetic analysis was performed to examine the genetic differences between the six populations: Africans, Ad-Mixed Americans, East Asians, Egyptians, Europeans, and South Asians. Multiple stages were required to preprocess the data and extract meaningful insights.

First, the data were sorted, annotated, and then merged using the “plyr” package v1.88^54^ in R to assure a standardized ID column format. Non-genotype columns have been eliminated, and the output has been converted to VCF format. BCFtools were used to further process the sorted file to ensure data accuracy.

The populations' genetic information was then extracted using principal component analysis (PCA). Using Plink v2.0^55,56^, an input bed file was generated, and variants were pruned based on a MAF of 0.05 as well as an indep-pairwise parameter of 50 5 0.5 (Window size in SNPs of 50, with a step of 5 SNPs to shift the window, and the r^2^ threshold to be 0.5) to assure independence between variants. This step is intended to eliminate closely related variants to maintain the accuracy of subsequent analyses.

The extracted PCA data offered eigen vectors and eigen values for 2614 samples, based on 1961 pruned variants. These elements captured the genetic diversity present in the populations and laid the foundation for further analysis.

The analysis was then transferred to the R for data visualization. The subpopulation listings of the populations were intersected with the Egyptian list to comprehend the overlap and shared variants. The “UpSetR” package v.1.4^57^, which generated interactive and flexible plots of intersecting sets, and an online tool from the Van de Peer Lab at the University of Ghent in Belgium (accessed via: https://bioinformatics.psb.ugent.be/webtools/Venn/) were used to generate a non-symmetrical Venn diagram. Using the "join" function from the R "plyr" package, the genomic data from the 1000 Genome and Egyptian genome files were combined to visualize the allele frequencies among the populations. This procedure of merging datasets enabled a comprehensive analysis of the genetic variation present in both datasets. Using the "pheatmap" package v1.0.12^58^, a heatmap was generated to visually depict the similarities and differences in allele frequency among the populations.

In addition, principal component analysis (PCA) was performed using the "prcomp" function in R to analyze the allele frequency discrepancies among populations. Using the "plot_ly" function from the "plotly" v4.10.1^59^ package, the first two components that accounted for a substantial portion of the cumulative variation were plotted. This additional visualization revealed the genetic distinctions among the populations based on their scores on the principal component.

Moreover, a genotypes PCA visualization was performed using R. The previously resulted eigenvalues and eigenvectors were used to calculate proportional variances, which were then displayed as a bar chart. To comprehend the role of geography and other demographic/clinical factors, the metadata of the 1000 Genome samples^60^ and Egyptian samples^42^ were utilized. The first two and three principal components were plotted against one another, providing a clearer picture of the genetic similarities and differences between the populations, and allowing the identification of prospective patterns in the data.

This thorough analysis and representation of genetic variation provided valuable insights into the diversity of the studied populations.

### Unique Egyptians’ single nucleotide variants (SNVs) and prediction of protein structural and functional damage

Additional research was conducted to learn more about the unique single nucleotide variants (SNVs) discovered in the Egyptian population. The Ensembl’s VEP was used to analyze the functional impact of these SNVs and predict the amino acid changes corresponding to them. The output of VEP was then filtered based on the genes of interest, with a particular emphasis on SNVs that were protein-coding and predicted to have amino acid alterations.

In addition, the AlphaFold database v2.0^61,62^ was used to acquire insight into the potential impact of identified SNVs on protein structure. This database contains predicted protein structures for protein-coding genes that have been filtered. The predicted structure in the PDB file format, as well as the anticipated amino acid change and its position, were input into the Missense3D database v1.5.4^63,64^. Missense3D utilizes computational algorithms to predict potential missense structural modifications resulting from amino acid substitutions.

This study aimed to shed light on the potential functional implications of genetic variations found in the Egyptian population by combining a unique SNV analysis with the prediction of protein structural damage.

### Supplementary Information

Supplementary Information 1.

Supplementary Information 2.

Supplementary Information 3.

Supplementary Information 4.

## Data Availability

1k genome variants data was obtained from the December 2021 Ensemble database. Diabetes data was accessed from: (http://dec2021.archive.ensembl.org/Homo_sapiens/Phenotype/Locations?oa=EFO:0001360). Obesity data was accessed from: (https://dec2021.archive.ensembl.org/Homo_sapiens/Phenotype/Locations?ph=3816). Hypertension data was accessed from: (http://dec2021.archive.ensembl.org/Homo_sapiens/Phenotype/Locations?oa=EFO:0000537). Hyperlipidemia data was accessed from: (http://dec2021.archive.ensembl.org/Homo_sapiens/Phenotype/Locations?oa=HP:0003077). The Egyptian Genome VCF files were obtained from the European Genome-Phenome Archive under dataset ID EGAD00001006039: (https://ega-archive.org/datasets/EGAD00001006039).

## Code availability

The code to reproduce the pipeline from this work can be found at GitHub: (https://github.com/Mahmoudbassuoni/EGYREF-Metabolic).
