# Supplementary material for: A comprehensive analysis of genetic risk for metabolic syndrome in the Egyptian population via allele frequency investigation and Missense3D predictions
Source: Sci Rep. 2023 Nov 22;13:20517. doi: 10.1038/s41598-023-46844-z (PMC10665412; doi:10.1038/s41598-023-46844-z)
Supplement: Supplementary file 3 — Supplementary Information 3. [file 41598_2023_46844_MOESM3_ESM.pdf]

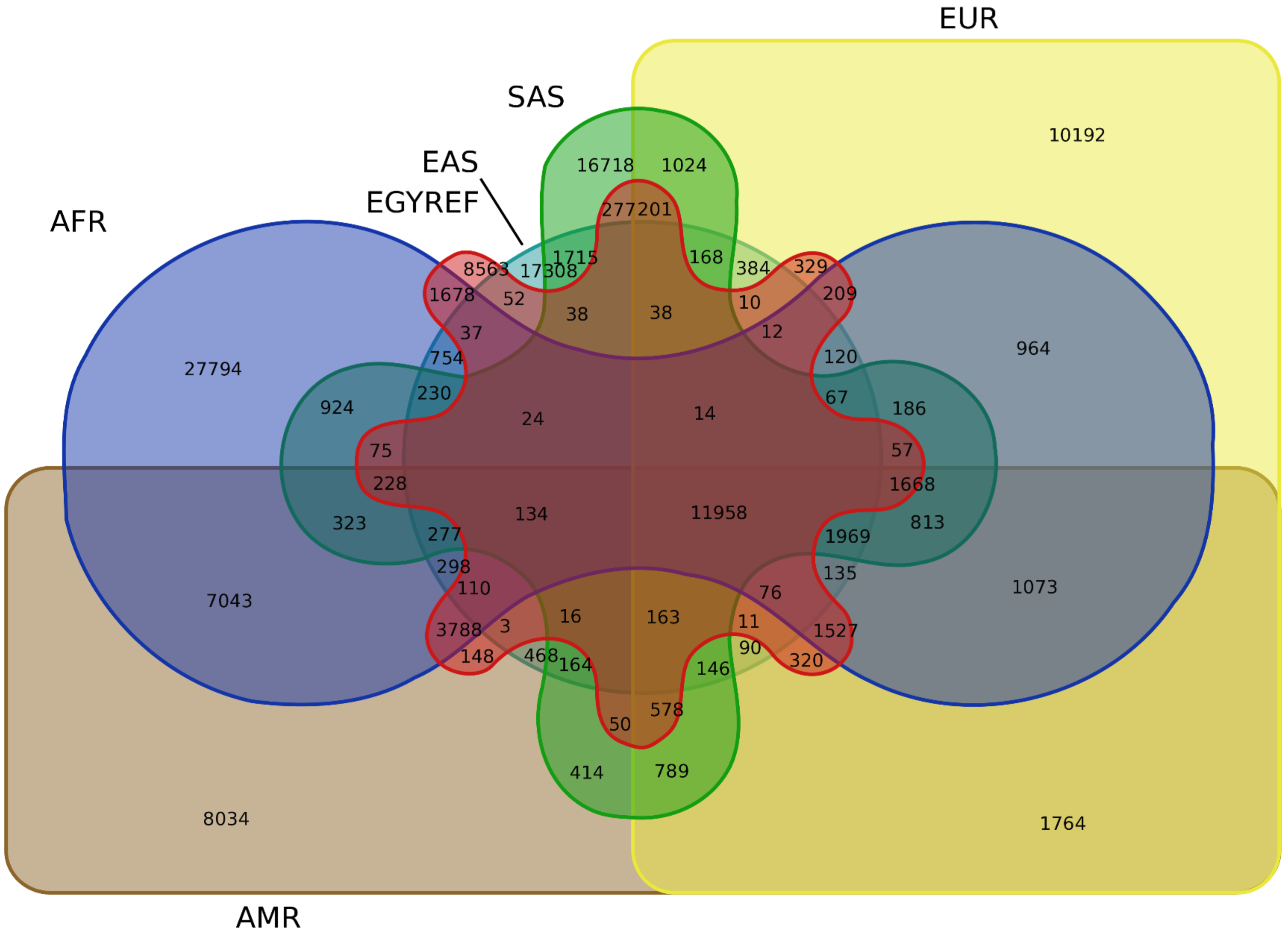

Supplementary figure S1: Non-symmetrical Venn diagram generated using the online tool from the Van de Peer Lab provides a graphical representation of the intersection between the different populations. The diagram consists of six circles representing the six populations included in the study. The overlapping regions between the circles show the number of variants shared between two or more populations. The diagram is non-symmetrical, which means that the size and shape of the overlapping regions between the circles are not necessarily the same for all possible combinations of populations. This reflects the fact that the genetic variation between the populations is not equally distributed and can result in different patterns of overlap between them.

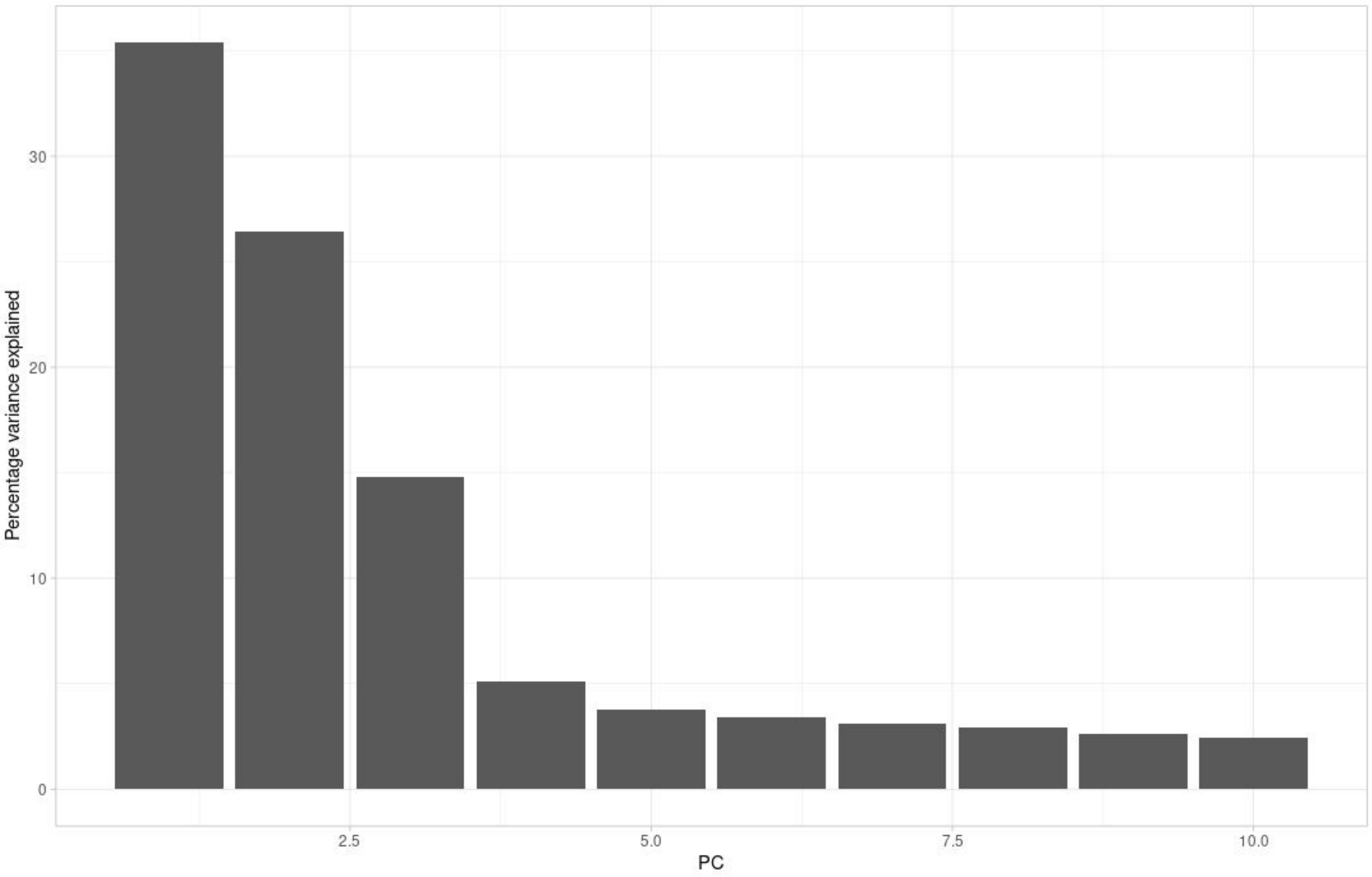

Supplementary figure S2: The bar plot shows the proportional variances of the principal components resulting from the principal component analysis (PCA) of the genetic variation dataset. The PCA resulted in eigenvalues and eigenvectors, which were loaded and analyzed to determine the relative contribution of each principal component to the overall variation in the dataset. The bar plot provides a clear visual representation of the distribution of the variation across the principal components, with each bar representing the proportional variance of a specific principal component. The height of each bar reflects the contribution of that principal component to the total variation in the dataset, allowing for easy identification of the most significant sources of variation.
